# Advances in Huntington’s Disease Biomarkers: A 10-Year Bibliometric Analysis and a Comprehensive Review

**DOI:** 10.3390/biology14020129

**Published:** 2025-01-26

**Authors:** Sarah Aqel, Jamil Ahmad, Iman Saleh, Aseela Fathima, Asmaa A. Al Thani, Wael M. Y. Mohamed, Abdullah A. Shaito

**Affiliations:** 1 Medical Research Center, Hamad Medical Corporation, Doha P.O. Box 3050, Qatar; sarahhaqel@gmail.com; 2 Medical Education, Hamad Medical Corporation, Doha P.O. Box 3050, Qatar; jamilnidal@gmail.com; 3 Biological Science Program, Department of Biological and Environmental Sciences, College of Art and Science, Qatar University, Doha P.O. Box 2713, Qatar; imanesaleh@qu.edu.qa; 4 Biomedical Research Center (BRC), QU Health Sector, Qatar University, Doha P.O. Box 2713, Qatar; af1907403@qu.edu.qa (A.F.); aaja@qu.edu.qa (A.A.A.T.); 5 Department of Biomedical Sciences, College of Health Sciences, QU Health Sector, Qatar University, Doha P.O. Box 2713, Qatar; 6 Department of Basic Medical Sciences, Kulliyyah of Medicine, International Islamic University Malaysia (IIUM), Kuantan 50728, Malaysia; waelmohamed@iium.edu.my; 7 Clinical Pharmacology Department, Menoufia Medical School, Menoufia University, Shebin El-Kom 32511, Egypt; 8 College of Medicine, QU Health Sector, Qatar University, Doha P.O. Box 2713, Qatar

**Keywords:** Huntington’s disease, biomarkers, rare diseases, neurodegenerative disorders, premanifest HD, preHD, neurofilament light chain (NfL), microRNAs (miRNAs), diffusion tensor imaging (DTI)

## Abstract

Neurodegenerative disorders (NDs) cause progressive neuronal loss and are a significant public health concern, with NDs projected to become the second leading global cause of death within two decades. Huntington’s disease (HD) is a rare, progressive ND caused by an autosomal-dominant mutation in the huntingtin (*HTT*) gene, leading to severe neuronal loss in the brain and resulting in debilitating motor, cognitive, and psychiatric symptoms. Given the complex pathology of HD, biomarkers are essential for performing early diagnosis, monitoring disease progression, and evaluating treatment efficacy. However, the identification of consistent HD biomarkers is challenging due to the prolonged premanifest HD stage, HD’s heterogeneous presentation, and its multiple underlying biological pathways. This study involves a 10-year bibliometric analysis of HD biomarker research, revealing key research trends and gaps. The study also features a comprehensive literature review of emerging HD biomarkers, concluding the need for better stratification of HD patients and well-designed longitudinal studies to validate HD biomarkers. Promising candidate wet HD biomarkers— including neurofilament light chain protein (NfL), microRNAs, the mutant HTT protein, and specific metabolic and inflammatory markers— are discussed, with emphasis on their potential utility in the premanifest HD stage. Additionally, biomarkers reflecting brain structural deficits and motor or behavioral impairments, such as neurophysiological (e.g., motor tapping, speech, EEG, and event-related potentials) and imaging (e.g., MRI, PET, and diffusion tensor imaging) biomarkers, are evaluated. The findings underscore that the discovery and validation of reliable HD biomarkers urgently require improved patient stratification and well-designed longitudinal studies. Reliable biomarkers, particularly in the premanifest HD stage, are crucial for optimizing HD clinical management strategies, enabling personalized treatment approaches, and advancing clinical trials of HD-modifying therapies.

## Simple Summary

Huntington’s disease (HD) is a rare neurodegenerative disorder characterized by progressive neuronal loss, leading to debilitating motor and cognitive deficits. Despite advances, identifying reliable biomarkers for early detection and disease monitoring remains challenging. This study includes a 10-year bibliometric analysis and a comprehensive literature review of HD biomarkers, highlighting promising candidates such as genomic, miRNA, protein, metabolic, and inflammatory markers, as well as biomarkers related to diagnostic imaging and neuropsychological tasks. Among the molecular HD biomarkers, neurofilament light chain (NfL) is a promising “wet” biomarker for premanifest HD that is pending further optimization and validation. The conclusions underscore the need for longitudinal studies to validate these biomarkers and standardized methods, especially for HD staging and patient stratification. Overcoming these challenges could transform HD care by enabling earlier detection, improved disease monitoring, better design of HD clinical trials, and personalized therapies.

## 1. Introduction

### 1.1. Huntington’s Disease: An Overview

Among the neurodegenerative disorders (NDs), Huntington’s disease (HD, OMIM 143100, https://omim.org/entry/143100, accessed on 19 January 2025) is the primary focus of this review. HD is a rare, monogenic, progressive, adult-onset, autosomal-dominant neurodegenerative disorder first described in 1872 by Dr. George Huntington. The disease results from mutations in the *huntingtin* (*HTT*) gene, first located in 1993 to chromosome 4p16.3 [1]. The *HTT* gene mutations, marked by a dynamic and variable expansion in the number of cytosine–adenine–guanine (CAG) repeats in exon1 of the *HTT* gene, cause the synthesis of abnormal huntingtin (HTT) protein. The expansion of CAG triplet repeats produces a toxic HTT protein with expanded polyglutamine (polyQ) amino acids [2]. The CAG repeat length has an inverse relation to the age of onset. Individuals with CAG repeat lengths between 8 and 26 are normal, while CAG repeat lengths of 27–35 are recognized as intermediate alleles that do not lead to the disease in the carrier but may increase to a pathogenic length in the progeny following germline transmission. CAG repeat lengths between 36 and 39 may express the disease with low penetrance, whereas repeat lengths exceeding 40 are fully penetrant and pathogenic [1,2,3,4].

Huntington’s disease is a rare neurodegenerative disorder with an estimated incidence of 0.38 per 100,000 person-years and a global overall prevalence of 2.71 cases per 100,000 individuals [3]. Though rare, its prevalence varies greatly (more than 10-fold) across geographical areas and populations [5,6]. Its highest prevalence is recorded within the Western/ White population (5–12 cases/100,000 individuals) [1]. HD has a higher prevalence in the USA (4.1–8.4/100,000 people) [3,5] and Europe (1.63–9.95/100,000 individuals) [7]. HD prevalence is lower in Asia (0.5 to 1.5 cases per 100,000 individuals) [5] and Africa (0.25 cases per 100,000 individuals) [3], although it increases in areas where intermarriage with Western individuals occurs [5]. China has the lowest HD prevalence (0.25 cases per 100,000 people). In Japan, the prevalence of the disorder is 0.5 cases per 100,000 people [5]. Huntington’s disease’s rarity becomes evident when compared with other NDs. For instance, in the USA, 9.92 million Americans suffer from mild cognitive impairment due to Alzheimer’s Disease compared with merely 30,000 HD patients [8]. NDs impose a burden on governments worldwide; in the USA alone, the mean total annual cost per patient with early to late-stage HD ranges from USD 6113 to USD 27,904 among commercially insured patients [9].

There is a need for consistent and reliable classification of HD disease in the period between birth and definitive clinical diagnosis, which now depends mainly on the diagnosis of motor functions [10]. Predictive and prenatal testing provides early opportunities for identifying HD gene carriers, guided by ethical frameworks and regional laws. These tests require pre-test counseling to support informed decision-making and address the significant implications for at-risk individuals and families [11]. HD is commonly classified into three stages: presymptomatic, prodromal, and manifest. In addition, HD stages before disease manifestation are broadly termed the premanifest or preHD stage. In the presymptomatic stage, individuals carry the CAG expansion mutation but exhibit no signs or symptoms related to HD. The prodromal stage includes individuals with the CAG expansion who display nonspecific or possible motor abnormalities on examination and subtle yet clear cognitive changes [12]. In today’s clinical guidelines, HD diagnosis relies on established motor, behavioral, and cognitive symptoms, all of which manifest after the disease has already proceeded [10]. In fact, the preHD or premanifest stage is prolonged, where manifest HD and the manifestation of unequivocal motor signs usually takes place between ages 30 and 50 [13]. Currently, the 1999 Unified Huntington’s Disease Rating Scale (UHDRS; UHDRS^®^’99), which relies on the diagnostic confidence level (DCL), is most commonly used for the clinical motor diagnosis of HD. DCL scores range from zero to four, with DCL4 representing a 99% confidence level in the physician’s diagnosis that the movement deficits are caused by HD. However, DCL4 marks a late disease stage and is inadequate for identifying the disease onset or detecting early changes that may precede observable clinical symptoms by decades [10]. Given the prolonged preHD stage, clinical staging criteria may not detect subtle changes in the preHD stage, underscoring the need for reliable biomarkers to facilitate early diagnosis and intervention [14]. Furthermore, HD progression prior to reaching DCL4 lacks standardization, with inconsistent terminologies—such as presymptomatic, premanifest, or prodromal—used across studies. This inconsistency has led to challenges in standardizing HD staging for participants in clinical trials and observational studies [10,14]. Therefore, research efforts have aimed to develop HD staging systems that accurately classify HD from birth till clinical diagnosis. Recently, the Huntington’s Disease Integrated Staging System (HD-ISS) has been proposed to offer a complementary framework of HD diagnosis. This system includes biomarkers staging and could refine our understanding of HD progression. The HD-ISS classifies *HTT* mutation carriers, supported by quantitative neuroimaging, cognitive, and functional markers, into research-purpose cohorts based on predicted disease progression. Unfortunately, none of the available HD wet biomarkers satisfied the criteria to be considered as classification landmarks for the HD-ISS. In the HD-ISS, Stage 0 encompasses mutation-positive individuals with no detectable clinical signs of disease, Stage 1 captures early biological changes, Stage 2 includes patients with noticeable clinical symptoms, and Stage 3 reflects functional decline [10].

**Figure 1:**
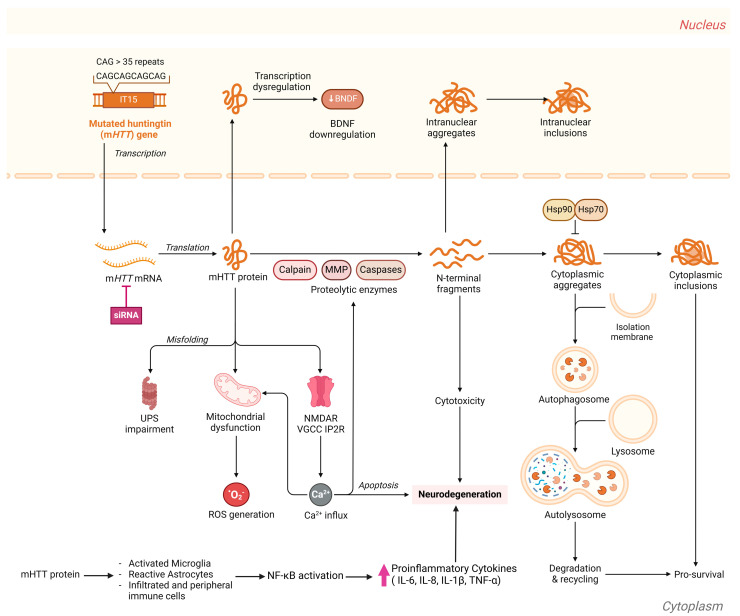
The intricate molecular pathways and mechanisms underlying pathology of Huntington’s disease.

Individuals with HD usually lead a normal life until the prodromal stage initiates. Symptoms of the disease generally appear in middle age, although it can present at any time from infancy to old age [15]. The first manifestations of neurological symptoms include a change in personality, depression, anxiety, restlessness, and deficits in social cognition, leading to stigmatization. In addition to difficulties with detail retention, patients often experience learning, organization, and task-planning challenges. During this stage, diagnosis is typically established and symptoms become progressively worse, ultimately resulting in deteriorating speech capabilities [16]. HD symptoms also encompass weight loss, which is attributed to the increased caloric demand resulting from incessant choreiform movements [1]. The main neuropsychiatric features of HD include the deterioration of motor functions encompassing involuntary irregular and unpredicted muscle movement, known as chorea, and involuntary muscle contractions, known as dystonia, in addition to deterioration in coordination skills, cognitive decline, and behavioral changes. As motor and cognitive functions decline, complications such as falls, dysphagia, or aspiration may lead to fatal outcomes. Typically, patients have a survival span of 15 to 20 years following diagnosis [17]. The progression of the disease profoundly impacts both patients and their families, necessitating comprehensive support that includes medical, psychological, and social aspects [1,18]. The complex and progressive HD pathophysiology is underpinned by intricate molecular and cellular processes and signaling pathways (Figure 1).

### 1.2. Challenges in HD Diagnosis and Treatment

The mutant HD gene produces a protein with a toxic gain of function due to polyQ expansion [19]. Mutant huntingtin proteins (mHTTs) with extended polyQ repeats undergo protein misfolding, leading to the accumulation of unmanageable protein aggregates, overwhelming cellular protein degradation via proteasomes and autophagic vacuolization [20]. Aggregation of mHTT, primarily within the striatum, which is important for coordination and motor functions, impacts many nuclear and cytoplasmic proteins involved in vital functions such as transcription regulation, apoptosis, vesicular trafficking, cellular metabolism and mitochondrial function, autophagy, neurotransmitter release, and axonal transport [21,22]. Binding of mHTT aggregates to essential transcription factors causes extensive gene expression alterations. mHTTs affect mitochondrial function, causing inefficient energy production, which elevates oxidative stress and exacerbates injury of vulnerable neurons [23]. Nevertheless, the exact neuronal function affected by mHTT misfolding and aggregation remains unclear [21]. Notably, there is evidence that m*HTT* mRNA is toxic and plays a role in the pathogenesis of the disease [24]. Studies have highlighted the vital role of HTT mRNA variations, particularly uninterrupted CAG repeat length, in influencing the onset and severity of HD. Disease progression correlates more strongly with the length of uninterrupted CAG repeats in the mRNA rather than with the length of polyQ in the mHTT protein [25,26,27].

Traditionally classified as a neuropsychiatric disease, recent research into the molecular mechanisms underlying HD suggests it is better described as a systemic illness, as autonomic symptoms often precede motor deficits by several years [15]. Beyond its well-documented neurodegenerative impacts, HD manifests in peripheral tissues, including skeletal muscle and liver, leading to metabolic dysfunctions [28]. Studies have reported mitochondrial impairments in both symptomatic HD patients and asymptomatic mutation carriers, indicating that mitochondrial dysfunction is an early feature of the disease [29]. The recognition of HD as a systemic disorder underscores the importance of comprehensive therapeutic approaches that address both central and peripheral manifestations. Targeting metabolic dysfunctions in peripheral tissues, alongside neurodegenerative processes, may offer new avenues for treatment interventions aimed at alleviating the multifaceted symptoms of HD [30]. This shift in understanding highlights the complexity of HD, both in its clinical presentation and underlying pathophysiology. Despite significant research developments over the last two decades, there has been limited advancement in medical treatments for HD [18].

Several approaches are proposed to develop HD drugs for therapy or management, including gene editing of the m*HTT* gene, interfering with or knocking down m*HTT* gene expression, and decreasing mHTT protein accumulation and/or aggregation using small molecule inhibitors and molecular chaperones [22]. Various therapeutic strategies, including antisense oligonucleotide (ASO) therapy, have been explored, but none have been proven effective in halting disease progression [31,32,33]. The recently introduced genetic editing techniques are also being investigated for HD gene therapy; for example, CRISPR/Cas9-induced double-strand breaks could cause CAG repeat contraction in the *HTT* locus [34], and an RNA-targeting CRISPR–Cas13d system was shown to mitigate HD-related deficits in a mouse model of HD by improving motor coordination, inhibiting striatal atrophy, and decreasing mutant HTT protein aggregates [32]. Another therapeutic strategy aims to enhance autophagy to aid cells in clearing mHTT aggregates and alleviate mitochondrial dysfunction [22]. Of note, the complexity, heterogeneity, and variability of HD manifestation implies that managing HD should be intricate, multifaceted, and tailored to each patient’s needs, often requiring a combination of pharmacological and non-pharmacological interventions [35].

HD remains relatively understudied compared with other neurodegenerative disorders due to its rarity, which limits the number of patients available for clinical studies. For instance, a one-year PubMed search yields approximately 1200 publications on HD compared with 16,400 on Alzheimer’s disease and 9900 on Parkinson’s disease. This lack of extensive research has resulted in a limited understanding of HD pathophysiology, presenting challenges in diagnosis and in the development of effective treatments. Consequently, current therapeutic approaches remain primarily symptomatic [36]. Compounding these challenges is the complexity and pleiotropy of HD clinical symptomatology, along with variations in cellular and neurochemical changes in the brain [37]. Studies suggest that the variability in symptoms cannot be fully explained by differences in CAG repeat numbers alone [38,39]. Somatic instability and genetic variations of DNA repair genes, such as MLH1 and MSH3, were shown to contribute to this variability. MLH1 drives somatic expansion and significantly affects disease onset age and the course of HD. Somatic expansion is not restricted to the brain but also occurs in peripheral tissues. This finding provides avenues to develop HD therapeutics that targets both mHTT toxicity and somatic instability and DNA repair mechanisms [40,41,42]. Additionally, genetic modifiers, such as MSH3, PMS1, PMS2, FAN1, and ATXN3, can impact the rate of CAG repeat expansion and the age of disease onset, contributing to differences in the rate of HD progression [41,43].

Adding to the complexity of HD symptoms, brain atrophy and structural disruptions occur during the prodromal phase before overt symptoms manifest. This period is characterized by intricate molecular and cellular changes that remain poorly defined [38]. Nevertheless, the premanifest stage offers a valuable window to explore novel therapeutic interventions that could delay HD progression before symptoms manifest [44]. These facts substantiate the search for new robust and sensitive diagnostic and prognostic HD biomarkers to improve knowledge of the disease pathophysiology and its underlying mechanisms in order to better predict HD pathogenesis, progression, and age of symptoms onset; and to explore patient response to potential therapies.

This study aims to perform a comprehensive bibliometric analysis of research on biomarkers in Huntington’s disease over the past decade. By examining publication trends, this study seeks to identify significant progress and research gaps, providing insights into the evolution of biomarker research. This study also examines the literature for biomarkers relevant HD at the different stages, including the preHD stage. These findings will provide insights into the evolution of HD biomarker research, reveal opportunities for future investigations, and offer recommendations to enhance understanding and development of biomarkers for effective diagnosis, progression monitoring, and therapeutic evaluation of emerging HD therapies.

## 2. Methods

This study includes a 10-year bibliometric analysis of the research on the development of HD biomarkers and a literature review of the potential HD biomarkers.

### 2.1. Bibliometric Analysis

Publications related to biomarkers of HD and published between 2014 and 2024 were sourced from PubMed through the open source Bibliometrix package version 4.3.1 in R software [45,46]. Bibliometrix, an open-source R package widely utilized by researchers for bibliometric analysis, offers comprehensive tools for quantitative analysis of scientific publications, including data collection, preprocessing, visualization, and statistical modeling [45,46]. The search strategy included the following terms: (“biomarkers” [All Fields] OR “biomarkers” [MeSH Terms] OR “biomarker” [All Fields]) AND “Huntington’s disease” [Title/Abstract] AND “English” [Language] AND “journal article” [Publication Type] AND (“1 January 2014” [Date-Publication]: “31 December 2024” [Date-Publication]). The search, conducted on 29 August 2024, covered various document types, such as original research articles, reviews, and conference proceedings, and was filtered to include only articles published in English.

### 2.2. Literature Review

The literature review was conducted using PubMed and Scopus to identify emerging and established biomarkers for Huntington’s disease (HD). The search strategy included terms such as “Huntington’s disease”, “biomarkers”, and related keywords. Articles published were screened for relevance based on titles, abstracts, and keywords. Full texts were reviewed to extract data on potential HD biomarkers, including their diagnostic, prognostic, and therapeutic implications.

## 3. Results

### 3.1. Bibliometric Analysis

**Figure 2:**
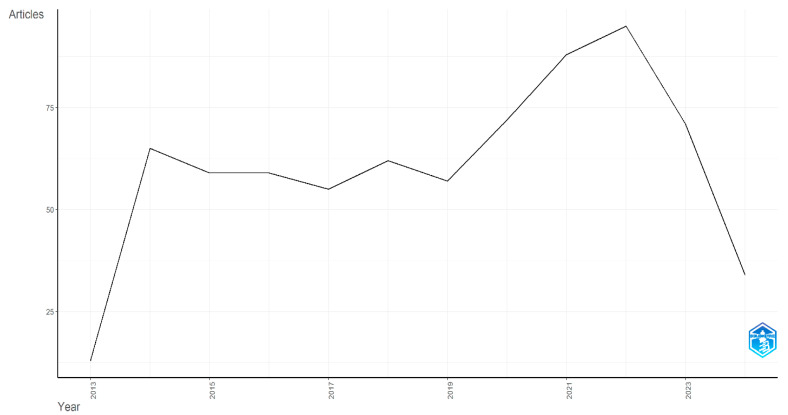
Trends of HD biomarker publications over time.

The analysis uncovered 730 articles related to biomarkers in Huntington’s disease, published between 2014 and 2024. The annual growth rate of publications in this field was 9.13% (Table 1). n total: 3959 authors from diverse countries contributed to these publications, with an average of seven co-authors per article. A significant rise in publications was observed from 2019 to 2022, with 2022 marking the peak at 95 articles (Figure 2).

**Figure 3:**
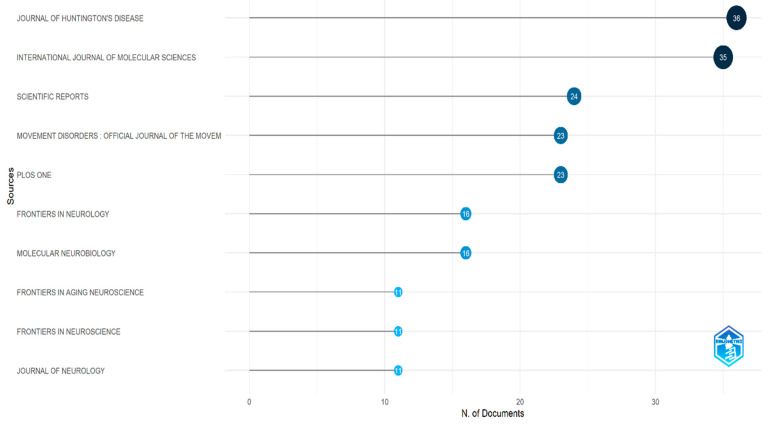
Journals where HD biomarkers research is published the most.

The articles were distributed across 319 different journals, with Journal of Huntington’s Disease publishing the highest number (36 articles), followed by the International Journal of Molecular Sciences (35 articles). A summary of the top 10 journals publishing the most articles in this research area is presented in Figure 3.

**Figure 4:**
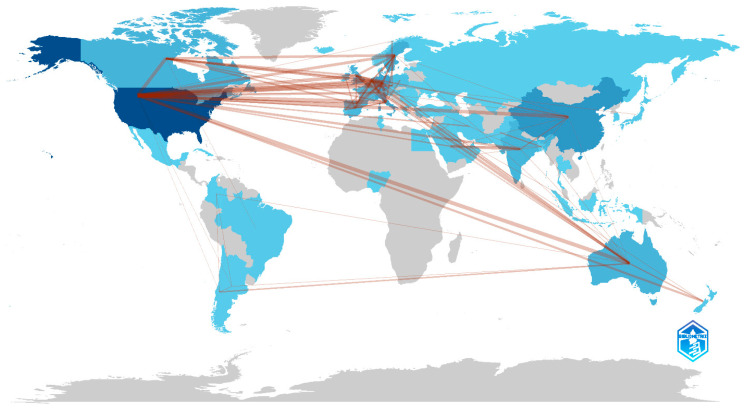
International collaboration network of research on HD biomarkers.

In terms of international collaboration, the strongest partnerships were observed between researchers in the USA and Germany, followed by collaborations between the USA and Australia and between the USA and Canada (Figure 4).

**Figure 5:**
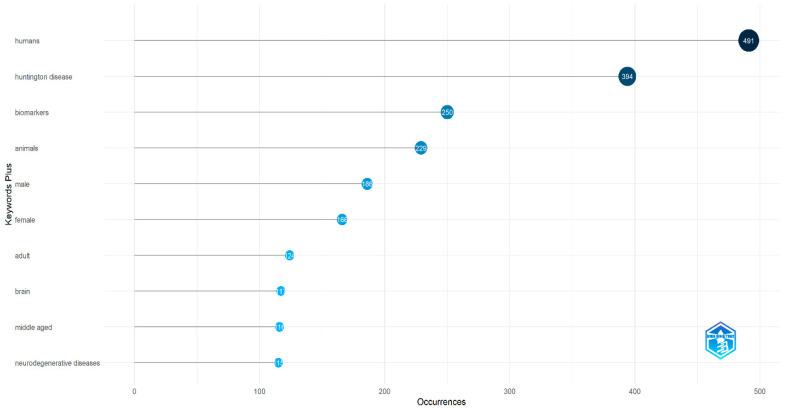
Most frequently used keywords in published studies investigating HD biomarkers.

**Figure 6:**
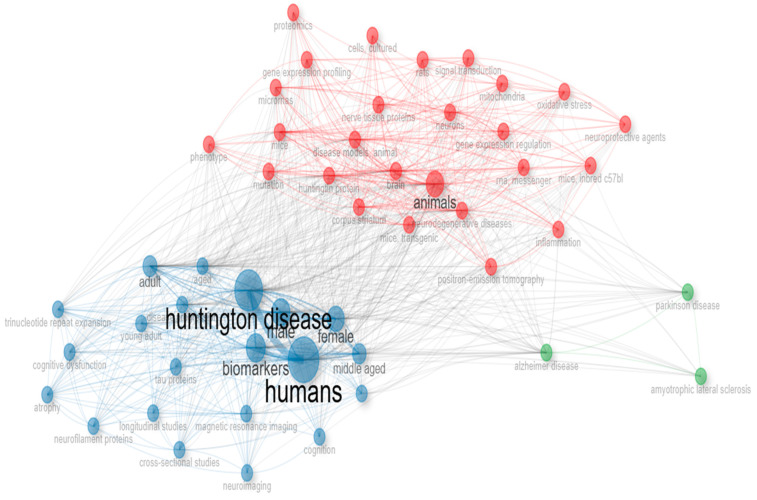
Network of the 50 keywords most frequently used in HD biomarkers published studies.

Through keyword analysis, we identified the top 10 most frequently occurring keywords. Of the 1684 keywords analyzed, “humans” appeared most frequently (491 occurrences), followed by “Huntington’s disease”, with 394 occurrences (Figure 5). Several other keywords also emerged as significant. This increase in keyword frequency mirrored the overall rise in publications. The co-occurrence network of the top 50 keywords is shown in Figure 6, where node size represents keyword frequency and node color indicates clusters and relationships. The keywords clustered into three main groups. The largest cluster, shown in blue, includes terms such as “humans”, “Huntington’s disease”, and “biomarkers”. The red cluster, which contains the most nodes, features terms like “animals”, “brain”, “neurodegenerative diseases”, and “disease models, animal”. The green cluster includes keywords like “Alzheimer’s disease”, “Parkinson’s disease”, and “amyotrophic lateral sclerosis”.

### 3.2. Huntington’s Diseases Biomarkers

Wu et al. proposed five reasons impeding the successful translation of promising HD drug candidates. The complexity of the cellular and molecular changes during HD progression, including the lack of knowledge of the exact molecular mechanisms involved; the uncertainty about the bioactivity networks, interacting small molecules, and modes of action of potential HD drug targets; the inadequate characterization of existing HD models and the shortage of efforts to develop more suitable disease models; and the need for reliable and sensitive biomarkers of HD onset and premanifest HD, apart from diagnosis of individuals carrying mHTT [47]. The length of CAG repeats in the mHTT gene can only broadly predict that a higher number of CAG repeats predicts earlier disease onset. Nevertheless, the age of disease onset of two individuals with the same CAG repeat length may differ by tens of years, posing serious challenges for the evaluation of potential therapy [48]. These facts substantiate the search for new robust and sensitive diagnostic and prognostic HD biomarkers to improve knowledge of the disease pathophysiology and its underlying mechanisms, better predict HD pathogenesis, progression, and age of symptoms onset, and explore patient response to potential therapies. These biomarkers are especially needed for the diagnosis and/or prognosis of changes in the premanifest HD stage before overt clinical motor deficits ensue. Aligning with this discussion, most of the research on HD biomarkers has aimed to discover reliable biomarkers that could predict the time to onset of clinical signs (phenoconversion) or monitor disease progression [49].

Table 2 lists biomarkers relevant to HD research. The relevance of these biomarkers is further discussed in the following sections.

In addition to biomarkers listed in Table 2, an array of HD biomarkers is emerging. These candidate biomarkers include reduction of kynurenine oxidative stress marker in preHD and increased conversion of tryptophan to kynurenine in late-stage HD [50,51]; leukocyte telomere length (LTL) [52,53], which is significantly shorter in preHD; the DNA double-strand breaks marker, histone variant pγ-H2AX, in peripheral blood mononuclear cells (PBMCs) as a reversible biomarker in preHD [54]; remarkable reduction of blood melatonin levels as HD progresses [50,55]; CSF levels of proenkephalin and prodynorphin, peptides mostly secreted by medium spiny projection neurons (MSNs) of the striatum due to dopaminergic signaling, were significantly reduced in HD patients in correlation with disease severity [53,56,57,58]; plasma GFAP levels were significantly increased in Chinese m*HTT* carriers and significantly correlated with disease burden and decline in motor function scores [59]; and dynein light chain Tctex type 1 (DYNLT1) levels in the whole blood was downregulated considerably at different stages of HD [60]. Furthermore, changes in the plasma metabolome, such as alterations in tryptophan, tyrosine, and purine metabolic pathways [50,61], and increased blood levels of 8-OHdG at HD onset [50], are further reported as candidate HD biomarkers. Recently, alterations in microbiome profiles have been documented during the course of HD progression [50], as well as between HD patients and healthy controls [50,62]. In confirmation, similar changes in microbiome profiles were reported in mouse models of HD [50].

Biomarkers of HD need further investigation and/or validation. For example, there are contradictory reports on the increase of 8-OHdG blood levels at HD onset, and Borowsky et al. confirm that plasma concentration of 8OHdG is not a biomarker of HD state or progression [63]. In alignment, a systematic review and critical appraisal scheme validating the ability of HD biomarkers to support the development of disease-modifying therapies reported that the methodological quality of existing research on biomarkers for HD progression is low, highlighting the need for better-designed studies to discover reliable HD biomarkers [49].

**Table 2:** Summary of biomarkers in Huntington’s Disease research and their characteristics.

	Biomarker	Source	Clinical Utility	Advantages and Limitations	References
**Genomic Biomarkers**	*HTT* gene mutation/CAG repeat expansion	Blood, Saliva	Disease diagnosis; predictive testing; prenatal testing	- High specificity and sensitivity; Can identify risk before clinical symptoms appear - There is a nonlinearity and plateau effect at extreme repeat lengths which limits the ability of mHTT alone to predict disease severity [64]	[64,65,66,67,68]
**Protein Biomarkers**	Mutant huntingtin (mHTT) protein	Blood, CSF	Disease progression monitoring, therapeutic targeting	- Wet biomarker - Directly linked to HD pathology - Limited sensitivity and challenging quantification	[65,69,70,71,72,73,74]
	Neurofilament light chain (NfL)	Blood, CSF	Neurodegeneration marker; disease progression marker; Can be used in preHD	- Wet biomarker - Reflects neuronal damage - May indicate other neurodegenerative diseases	[75,76,77,78,79,80,81,82]
	Brain-derived neurotrophic factor (BDNF)	Blood, salvia, CSF	Potential early disease marker	- Wet biomarker - Non-invasive sampling - Inconsistent findings among studies, BDNF is produced in multiple tissues, complicating the interpretation of peripheral levels	[44,83,84,85,86,87,88,89]
	Tau protein	Blood, CSF, skin tissue	Elevated levels of phosphorylated tau (p-tau) in plasma correlate with HD severity, aiding in staging the disease, abnormal tau accumulation in skin tissue may serve as an early indicator of HD.	- Wet biomarker - Non-invasive sampling, standardized methods exist for measuring tau levels in CSF and blood, facilitating clinical implementation - Lack of specificity, inconsistent findings	[90,91,92,93]
	IL-6	Blood, CSF	Inflammation monitoring, disease progression	- Wet biomarker - Indicates neuroinflammation, potential for early intervention - Non-specific, can be elevated in other conditions	[94,95,96,97]
**Non-coding RNA Biomarkers**	microRNAs (miRNAs)	Blood, CSF, brain tissue	Gene expression regulation, disease state indicators	- Non-invasive, early detection potential - Requires complex analysis; variability in expression	[98,99,100,101,102,103]
**Metabolic Biomarkers**	Uric acid	Blood, saliva	Potential predictor of disease progression in HD	- Wet biomarker - Associated with slower progression of functional decline in HD - Gender differences in UA levels may complicate interpretation	[104,105,106,107]
	24S-Hydroxycholesterol (24S-OHC)	Brain-derived; measurable in plasma	Reduced plasma levels of 24S-OHC correlate with disease progression, Alterations in 24S-OHC levels may help identify premanifest HD individuals	- Wet biomarker - Reflects brain cholesterol metabolism, non-invasive measurement - Altered 24S-OHC levels are observed in various neurodegenerative disorders, not exclusively in HD, which may limit its specificity as a biomarker, limited longitudinal data	[44,108,109,110,111,112]
**Neurophysiological Tasks Biomarkers**	Motor tapping	Speeded tapping tasks measuring the number and rhythm of taps within a set time frame.	Identifies subtle motor deficits in premanifest HD individuals, Tracks motor decline over time.	- Provides objective data on motor function, detects changes not captured by traditional motor scales - Performance can be influenced by factors like motivation and attention, limited specificity as it may not distinguish between different motor disorders.	[113,114,115,116,117]
	Speech biomarkers	Acoustic analysis of speech patterns, including articulation rate, pause duration, and prosody.	Tracks progression of speech impairments correlating with motor and cognitive decline, Associates speech changes with genetic markers like CAG repeat length.	- Quantitative analysis, early indicator - Influenced by individual differences, needs specialized software and expertise for analysis, speech changes may occur in other neurodegenerative diseases, necessitating comprehensive assessments	[118,119,120,121,122,123]
	Event-related potentials (ERPs)	Measurement of brain’s electrical response to specific sensory, cognitive, or motor events using EEG	Cognitive function assessment, disease progression monitoring	- Captures rapid neural responses, safe for repeated measurement, reduces reliance on subjective assessments - Technical expertise required, less precise in localizing neural sources compared to other imaging techniques	[124,125,126,127,128,129,130]
	Electroencephalography (EEG)	Brain	Identifies neural activity alterations in premanifest and manifest HD stages.	- Safe and painless procedure, more affordable compared to other neuroimaging techniques. - Less precise in localizing specific brain regions; needs skilled personnel for accurate interpretation; External factors can affect data quality	[131,132,133,134]
**Imaging Biomarkers**	MRI	Brain	Structural changes, disease progression	- Non-invasive, visualizes brain changes - Expensive, requires specialized equipment are needed	[135,136,137,138]
	PET scan	Brain	Functional brain imaging, neurotransmitter activity	- Provides functional data, specific for HD - Expensive, limited availability, requires radioactive tracers	[139,140,141,142]
	Diffusion tensor imaging (DTI)	Brain	Early detection, disease progression monitoring	- Non-Invasive, sensitive to microstructural changes - Susceptible to differences in imaging protocols and analysis methods, potentially affecting reproducibility; limited availability	[143,144,145,146,147]

#### 3.2.1. Genomic Biomarkers

##### HTT Gene Mutations/CAG Repeat Expansion

The genetic detection of CAG repeat expansions in exon 1 of the HTT gene (m*HTT*) is the definitive molecular diagnosis for HD. One significant benefit of m*HTT* as an HD biomarker is its ability to diagnose HD incidence, predict the disease onset, and help monitor its progression many years before symptoms appear, particularly in individuals with more than 39 CAG repeats. Diagnostic testing is conducted when a patient exhibits the characteristic motor symptoms of Huntington’s disease to confirm clinical diagnosis [65]. Predictive testing, on the other hand, is performed in asymptomatic adults who are at risk of inheriting the *HTT* gene mutation, allowing for the anticipation of disease onset before clinical symptoms emerge. This predictive capability allows for the early identification and monitoring of at-risk individuals, enabling researchers to study the preclinical phase of neurodegeneration in detail. Consequently, it provides crucial insights into the reasons behind the variability in the age of onset [148] and the progression of motor and cognitive deficits [149], facilitating the development of targeted early interventions and personalized treatment plans. Longer repeats are associated with an earlier onset [150], particularly in the case of longer repeats (>60Q) [151]. Multiple studies examining various ranges of CAG repeat lengths indicate that variations in the number of CAG repeats account for 40–60% of the variation in HD onset age, with less correlation observed in individuals closer to the normal repeat range [152]. The CAG age product (CAP) score, defined as the product of age and excess CAG length, reflects both the length of CAG repeat expansion and the duration of its impact, i.e., the cumulative exposure of a carrier to m*HTT*. Autopsy findings confirm that CAP may serve as a reliable indicator of disease severity and pathology in HD patients [153]. This tool could help researchers and clinicians to better understand disease progression and to design and interpret clinical trials more effectively. Several foundational HD clinical trials, such as the Enroll-HD platform [154], a large, international, well-characterized, research-oriented cohort with more than 25,000 participants) with associated biological samples and clinical data, have used the CAP score; Enroll-HD has supported several HD studies such as HDClarity, ImageClarity, iMageHTT, FOCUS-HD, PACE-HD, and Origin-HD [154].

#### 3.2.2. Wet HD Biomarkers

##### Non-Coding RNA Biomarkers

Recent studies on the changes in the profiles of non-coding RNAs in HD patients, particularly in the periphery (blood), have identified several micro-RNAs (miRNAs) that are differentially dysregulated in HD patients [44,50,101,155,156,157,158,159]. There are reports of the dysregulation of other non-coding RNAs in HD patients, for example, small nucleolar RNAs (snoRNAs), which are a class of small non-coding RNAs involved in the control of chemical modifications, alternative splicing, and post-transcriptional modifications of other RNAs. Higher levels of U13 snoRNA (SNORD13) were reported in HD patients compared with controls, indicating that this snoRNA may serve as a peripheral marker of HD; this is pending further validation [160].

##### MicroRNAs (miRNAs)

MicroRNAs (miRNAs) are small, conserved, non-coding RNAs consisting of RNA sequences of 20 to 30 nucleotides that regulate the expression of target genes post-transcriptionally, impacting crucial physiological and pathological processes [98]. miRNAs have emerged as potential biomarkers of NDs due to their abundance in biofluids, where they are typically protected within exosomes, micro-vesicles, apoptotic bodies, or protein complexes such as high-density lipoprotein [100]. miRNA availability in biofluids such as blood or CSF highlights their potential utility as peripheral biomarkers. Dysregulated miRNA expression patterns have been implicated in the etiology and progression of several polyQ diseases, including HD. Langfelder et al. demonstrated that alterations in miRNA expression are crucial to HD pathogenesis [102]. Extensive research has revealed altered miRNA expression in cellular models, mouse tissues and, importantly, in the brains of affected HD patients [44,50,99,100,101,155,156,157,158,159,161]. hsa-miR-34b was the first miRNA reported to be dysregulated in HD. Plasma hsa-miR-34b levels were increased in preHD and not in manifest HD patients or normal controls [162]. Later, dysregulation of several miRNAs has been implicated in the onset and progression of HD, offering valuable insights into the molecular mechanisms underlying HD and opportunities for the design of therapeutic and diagnostic strategies for the disease [44,50,99,100,101,155,156,157,158,159,161]. Hoss et al. identified five differentially expressed miRNAs (miR-10b-5p, miR-196a-5p, miR-196b-5p, miR-10b-3p, and miR-106a-5p) in the brain of HD patients. Brain miR-10b-5p expression in particular was strongly correlated with age of onset and extent of striatal involvement [155]. Another study tested the expression of 13 miRNAs in the peripheral leukocytes and identified miR-9* expression to be significantly reduced in HD patients versus healthy controls [157]. Díez-Planelles et al. compared the circulating miRNome (752 human mature miRNAs) in the blood and brain of symptomatic HD patients versus healthy matched controls and identified 168 cmiRNAs with altered expression in the symptomatic HD patients. HD patients’ blood levels of thirteen miRNAs (miR-877-5p, miR-223-3p, miR-223-5p, miR-30d-5p, miR-128, miR-22-5p, miR-222-3p, miR-338-3p, miR-130b-3p, miR-425-5p, miR-628-3p, miR-361-5p, and miR-942) were significantly increased. Expression of miR-122-5p was significantly decreased in the blood of HD patients, in correlation with the age of symptoms onset and UHDRS scale, whereas the increased miR-100-5p expression and decreased miR-641 and miR-330-3p expression in the blood of HD patients was in correlation with the total functional capacity (TFC) clinical scale [156].

Investigation of CSF miRNA levels of 60 participants in the PREDICT-HD study as potential biomarkers for prodromal HD identified six miRNAs—miR-520f-3p, miR-135b-3p, miR-4317, miR-3928-5p, miR-8082, and miR-140-5p—to be significantly elevated in prodromal HD compared with controls. These miRNA levels showed a progressive increase from control to low-risk stages and from low- to medium-risk stages, stabilizing in high-risk and diagnosed groups [101]. A most recent study aggregated and analyzed already published data of extracellular miRNA in the CSF and identified hsa-miR-361-3p to be significantly downregulated in HD patients compared with healthy controls [163].

Overall, the use of miRNAs as peripheral biomarkers of HD is promising but remains limited and inconsistent and needs future exploration and validation [44,103].

##### Protein Biomarkers

Mutant Huntingtin (mHTT) Protein

Advanced research in proteomics and metabolomics has been instrumental in identifying potential fluid biomarkers for Huntington’s disease. The presence of mHTT protein in biological fluids, such as blood and CSF, is a primary molecular biomarker for HD. mHTT protein is crucial in HD pathogenesis [164], leading to neuronal damage and death [69]. Quantifying mHTT in CSF aids in diagnosing the disease, assessing its severity, and monitoring the effectiveness of treatments [70]. It was demonstrated that mHTT protein levels in the CSF could correlate with disease stage, symptom severity, and markers of neuronal damage in people with HD and could serve as diagnostic biomarkers, accurately distinguishing between controls and HD mutation carriers [71,72,165].

Huntingtin protein (HTT) is present in extremely low amounts in CSF [73], requiring highly-sensitive sensitive assays for its detection. A cross-sectional study the mHTT protein levels in the blood and CSF of HD patients was conducted using single molecule counting (SMC) immunoassays and microbead-based immunoprecipitation combined with flow cytometry (IP-FCM) [72,165]. Both were specific in detecting mHTT protein [73]. mHTT protein levels were elevated in the CSF of HD patients compared with control subjects, providing the ability to differentiate between premanifest and manifest stages of HD [72,73]. Vauleon et al. developed an assay that could quantify mHTT protein in CSF, proposing that this assay could be reliably employed to support the development of mHTT-lowering therapies [166]. Fodale et al. further optimized the SMC assay to quantify HTT independent of polyQ length in the CSF of HD patients and controls, with high selectivity and specificity [167].

There is a limitation of measuring brain-derived mHTT protein in the periphery in the blood as there is a ubiquitous production of mHTT throughout the body, and distinguishing between central nervous system (CNS)-derived mHTT and its peripheral counterpart is challenging [74,82]. Sensitive assays based on homogeneous time-resolved Förster resonance energy transfer (HTRF) or the Meso Scale Discovery electrochemiluminescence immunoassay platform (MSD) were designed to approach this problem. They successfully detected mHTT in peripheral leukocytes or blood [50,74,168]. The levels of mHTT in the blood and saliva were also determined by several research groups, and they were different between HD patients and healthy controls [50,74,168,169].

##### Neurofilament Light Chain (NfL)

Neurofilaments, a type IV intermediate filament, are crucial for neuronal function, including maintaining neuronal structure and axonal integrity. They are predominantly expressed in myelinated axons. Neurofilaments are classified by their molecular weight into heavy (NfH), intermediate (NfM), and the most soluble light chain (NfL) proteins. Particularly, NfL, also abbreviated as NFEL or NFL, stands out as a marker of neuronal injury of NDs [170]. NfL is the most promising wet biomarker of HD and can be measured in cerebrospinal fluid (CSF), plasma, and serum [75,76,77]. Wet biomarkers, such as NfL, can facilitate clinical management and therapy development in HD as altered levels of NfL in CSF and blood were shown to be associated with HD onset, severity, and progression [78].

Additionally, NfL levels in blood have been shown to predict regional brain atrophy in HD. Byrne et al. demonstrated that elevated plasma NfL levels are associated with neurodegeneration and regional brain atrophy, particularly in the caudate nucleus and putamen, supporting NfL’s potential as a biomarker for disease progression in HD [79]. Furthermore, NfL plasma baseline levels were elevated in preHD and early-stage HD before symptom onset and were associated with a subsequent decline in cognition and functional capacity [50,79], suggesting its potential for predicting disease onset and progression in HD. Moreover, NfL protein concentrations in both CSF and plasma could segregate between premanifest and manifest HD [71,80,81,171], indicating their potential as early indicators of disease progression [82]. NfL is also beneficial in predicting early-onset versus late-onset HD patients [79].

Importantly, CSF NfL levels were elevated in the HD-YAS cohort of preHD individuals, who were around 24 years younger than the predicted clinical HD onset age. Elevated NfL levels correlated with biological and neurodegenerative changes in the preHD patients, proposing that NfL could be a sensitive biomarker in preHD individuals years before HD onset. This study further indicated that early neuronal damage appears even before clinical symptoms do (around 24 years earlier than HD onset age) and that this timepoint could be optimal for starting potential disease-modifying therapies in the future [172]. Relatedly, the HDClarity study showed that collecting CSF from lumbar punctures is safe and feasible in HD patients, further supporting the role of CSF biomarkers in HD biomarker research and facilitating the development of biomarkers like NfL for monitoring disease progression [173].

A semi-mechanistic model of concentration of NfL entering the CSF suggests that NfL concentration in CSF is a quantitative biomarker of neurodegeneration rate rather than extent [174]. Plasma NfL levels are associated with predicted years to clinical diagnosis, using the clinical motor diagnosis (CMD) and prognostic index normed (PIN) scores. Parkin et al. determined that plasma NfL concentrations ≥ 45.0 pg/mL could segregate *HTT* mutation carriers within ten years of CMD [171,175,176]. In a follow up study, Parkin et al. indicated that plasma NfL levels could be used to enrich cohorts of HD-ISS Stage 1 with HTT mutation carriers who are predicted to be less than, and within, 10 years until CMD [176]. This is significant because, to date, none of the wet HD biomarkers fit the stringent criteria to be recognized as a categorization landmark in the HD-ISS [10]. The findings of Parkins et al. indicate that plasma NfL levels could improve HD-ISS staging, especially for stages prior to CMD [176].

Many studies have reported increased NfL levels in HD patients compared with healthy controls [71,79,171,175,177]. Plasma NfL originates from injury of CNS neurons, and its levels should correlate with NfL levels in CSF [79]. Given the involvement of mHTT and NfL in HD incidence and progression, the simultaneous detection of both proteins was shown to provide additive insights in HD diagnosis and progression and determination of the therapeutic potential of candidate HTT-lowering drugs [71].

Notably, CSF NfL levels were a more sensitive biomarker than plasma NfL levels for monitoring disease progression in the early preHD stages, suggesting that the predictive power of plasma NfL in preHD patients far from disease onset may be limited compared with CSF NfL [172]. Nevertheless, a retrospective analysis of individuals from the TRACK-HD study showed that NfL concentrations in plasma significantly correlated with clinical and MRI findings, demonstrating its utility as a prognostic marker for neurodegeneration in HD [79]. TRACK-HD is a multicenter prospective observational biomarker study that assessed longitudinal data of 120 premanifest gene carriers (preHD), 123 early HD patients, and 123 matched healthy controls [178]. In contrast, Parkin et al. indicated that plasma NfL could indicate HD onset but not progression of symptoms [171]. These discrepancies need to be sorted out in future well-designed longitudinal studies that clearly segregate between preHD and manifest HD participants. Meanwhile, the use of plasma, and even CSF, NfL to support clinical trials of HTT-lowering drugs should be treated with caution [44].

##### Brain-Derived Neurotrophic Factor (BDNF)

BDNF, a neurotrophic factor crucial for neuronal growth and survival [179], has gained interest as a potential biomarker for HD. A study investigating BDNF levels in HD patients revealed that plasma BDNF levels were not significantly different across diagnostic groups, while salivary BDNF levels were notably lower in both premanifest and manifest HD patients compared with controls, indicating its potential as an early disease marker [85]. It is reported that mHTT protein could disturb the transcription and translation of BDNF, attenuating BDNF functions [180]. Relatedly, BDNF promoter IV DNA methylation was significantly altered in HD patients, with specific methylation sites being inversely correlated with anxiety and depression scores, suggesting its role in psychiatric symptomatology. However, the relationship between BDNF promoter methylation and anxiety requires further validation to be considered robust [85].

The alteration of BDNF blood levels in HD patients has produced controversial results. Earlier studies reported decreased blood BDNF levels in HD patients [86,87]. More recent studies, in contrast, present a more complex picture, with some studies finding no significant differences in blood-based BDNF levels across different stages of HD [88,89], and Ou et al. confirm that BDNF is not an HD biomarker as it was not associated with clinical scores or neuroimaging measures and had poor ability to discriminate m*HTT* carriers from healthy control or premanifest from manifest HD [89]. These disparities highlight the challenges in establishing BDNF as a consistent biomarker, possibly due to region-specific secretion properties and its complex origins. Collectively, the current research suggests a complex relationship between BDNF levels, DNA methylation, and HD progression, highlighting the necessity for continued exploration of the utility of BDNF as a biomarker for HD.

Tau Protein

Tau is a microtubule-associated protein (MAP) involved in neurogenesis, axon maintenance, and axonal transport. Tau has been extensively studied in NDs, including Alzheimer’s disease, Parkinson’s disease, and traumatic brain injury (TBI) [93,181,182,183,184]. Findings have demonstrated a strong association between tau and cognitive impairments, proposing that tau could be a promising HD biomarker [93]. Emerging evidence implicates tau in the pathology of HD, akin to tauopathies, suggesting that therapeutic strategies targeting tau dysfunction, ranging from small molecules to gene modulation approaches, could hold promise for addressing cognitive decline in HD [185]. Age-adjusted CSF tau levels were significantly elevated in m*HTT* gene carriers compared with healthy controls and correlated with disease progression. Specifically, CSF tau showed significant correlations with the total functional capacity and total motor score of the UHDRS scale, suggesting its potential as a biomarker for HD progression and monitoring therapeutic responses [186]. A study investigating the neuropathological, genetic, and clinical aspects of tau pathology in HD identified extensive aggregates of hyperphosphorylated tau in HD brains, some of which colocalized with mHTT. This colocalization was also present in cases of young-onset HD [91]. Another study has demonstrated that HD pathology is associated with increased presence and aggregation of tau, α-synuclein (α-Syn), and TAR DNA-binding protein 43 (TDP-43) in the brain, alongside abnormal phosphorylation of tau and altered splicing patterns of tau isoforms [187]. A recent study elucidated that tau exon 2 and exon 10 alternative splicing isoforms in HD putamen may serve as a biomarker or therapeutic target of HD, pending further investigation [188]. More recently, a study of a Korean HD cohort revealed that plasma levels of phosphorylated tau could serve as a biomarker of HD severity [189]. Skin phosphorylated tau was recently found to be elevated in manifest HD compared with premanifest HD and healthy controls and correlated with CAG repeat length, CAP score, motor function clinical scores, and neuroimaging data. The authors indicated that skin phosphorylated tau may serve as an HD biomarker and could be used to improve subject stratification, enhancing the distinction and validity of HD cohorts for clinical trials [92]. Nevertheless, further research is warranted to elucidate the diagnostic applications of these proteinopathies in HD.

Inflammatory Biomarkers

Inflammatory biomarkers play a significant role in HD pathogenesis and progression. mHTT protein profoundly impacts the immune system, driving inflammatory processes that are central to HD pathology. Microglial activation, which correlates with disease severity, occurs before the onset of clinical symptoms, highlighting the early involvement of the immune system. Abnormal immune activation is evident in the CSF and striatum of HD patients, where mHTT protein levels in monocytes and T cells strongly associate with disease burden and brain atrophy [190]. This inflammatory response, characterized by neuroinflammation, becomes an active contributor to the pathogenic process in HD [191]. Within the CNS, mHTT expression in immune cells promotes autonomous microglial activation and the secretion of proinflammatory cytokines, while in the periphery, it fosters a chronic state of systemic inflammation that drives disease progression [192]. Elevated plasma cytokine levels, including interleukin-6 (IL-6) and IL-8, further underscore the systemic nature of inflammation in HD [95]. The activation of the innate immune system is exemplified by impaired macrophage migration and complement factor deposition in the striatum, while dendritic cells (DCs), components of the adaptive immune system, contribute by priming T-cell responses and secreting inflammatory mediators. Notably, DCs may also harbor mHTT, emphasizing their potential role in perpetuating inflammation in HD [193].

Research indicates that plasma IL-6 levels are higher in moderate HD than in early-stage or premanifest HD, and a meta-analysis demonstrated significantly elevated levels in mutation-positive individuals compared with controls [94]. A recent study showed increased IL-6 levels in premanifest m*HTT* carriers about 16 years before the anticipated onset of motor symptoms [95]. Additionally, a meta-analysis has demonstrated significantly elevated plasma IL-6 levels in HD patients compared with healthy controls. The analysis included data from 469 HD mutation-positive individuals and 206 controls. Plasma IL-6 levels correlated with disease progression, showing significant increases between premanifest and manifest stages as well as between early and moderate stages of HD. Moreover, higher IL-6 levels were associated with more severe motor impairments and greater disability in daily activities, suggesting that IL-6 may be a viable biomarker for HD progression [96]. However, other studies have reported conflicting findings, with some showing no significant differences in IL-6 levels between manifest, premanifest HD, or healthy controls [88,194]. Similarly, while IL-6 and IL-10 levels were significantly higher in HD patients, no differences were observed for other inflammatory biomarkers such as C-reactive protein (CRP), complement component C3, interferon-γ (IFN-γ), IL-1, IL-2, IL-8, and tumor necrosis factor-α (TNF-α) [195].

Tumor necrosis factor-alpha (TNF-α) has been implicated in HD pathogenesis as a key mediator of neuroinflammation. Elevated TNF-α levels have been detected in the brains and plasma of HD patients, and they are believed to exacerbate neuronal damage through the activation of inflammatory pathways [196]. In preclinical HD models, inhibition of soluble TNF-α reduced neuroinflammation, neuronal toxicity, and motor impairments, underscoring its potential as a therapeutic target [197]. However, some studies have reported no significant differences in TNF-α levels between HD patients and controls, suggesting variability in findings and the need for further research to validate its role in HD progression and utility as a biomarker [195,196].

Additionally, cerebrospinal fluid (CSF) biomarkers have provided valuable insights into neuroinflammatory processes in HD. YKL-40, a marker of glial activation and neuroinflammation, has been observed to be significantly elevated in premanifest HD individuals. This rise in CSF YKL-40 levels reflects ongoing inflammatory processes even in the absence of clinical symptoms, underscoring its potential as a biomarker for early disease detection and progression monitoring [172]. Similarly, increased levels of proinflammatory cytokines like IL-17 and the presence of IL-17-producing Th17.1 cells in the CSF of premanifest individuals highlight the role of immune dysregulation in early HD pathology [198]. These findings suggest that inflammatory biomarkers, particularly those detected in CSF, could be critical in identifying early disease stages and monitoring progression.

Furthermore, elevated levels of interleukin-1β (IL-1β) have been observed in HD patients, contributing to neuroinflammatory processes. Activation of microglia and astrocytes in HD leads to the release of IL-1β, which can exacerbate neuronal damage [199]. In HD models, the NLRP3 inflammasome—a protein complex responsible for activating inflammatory responses—has been shown to increase IL-1β production. Inhibition of the NLRP3 inflammasome using selective inhibitors like MCC950 resulted in decreased IL-1β levels, reduced neuronal toxicity, and improved motor function in HD mice [200]. These findings suggest that IL-1β plays a significant role in HD progression and highlight the potential of targeting IL-1β-mediated pathways as therapeutic strategies.

Further evidence of inflammatory dysregulation in HD includes elevated plasma levels of cytokines such as IL-8, which were significantly associated with clinical motor scores [44]. The cellular immunophenotypes and high levels of secreted inflammatory cytokines and chemokines in peripheral circulation also serve as potential prognostic markers [95,97]. TGF-β1, another cytokine secreted in both the CNS and peripheral tissues, was positively correlated with cognitive impairment in early-stage HD, although no significant associations were found with disease duration, age of onset, or CAG repeat length [201,202].

Overall, while inflammation is a consistent feature of HD pathology, conflicting findings highlight the need for well-designed longitudinal studies with stratified patient cohorts to validate the clinical use of inflammatory biomarkers like IL-6, YKL-40, and IL-17 in HD diagnosis.

Metabolic Biomarkers

HD patients undergo metabolic disturbances, including weight loss, increased energy consumption, and altered cholesterol and amino acids metabolism. m*HTT* carriers with high body mass index (BMI) showed significantly slower progression to clinical symptoms [203]. The metabolic alterations of HD are accompanied by changes in the blood metabolome, offering avenues for the discovery of diagnostic, prognostic, or therapy-monitoring HD biomarkers. Changes in the plasma metabolome, such as alterations in tryptophan, tyrosine, and purine metabolic pathways [50,61]; and increased blood levels of 8-OHdG at HD onset [50], uric acid, and 24 (S) hydroxycholesterol (24OHC) are examples of candidate HD metabolomic biomarkers.

Uric Acid (UA)

Uric acid (UA) is a primary natural antioxidant in human blood produced from purine metabolism. While UA is known for its role in conditions like gout, it also influences the CNS and has been associated with neurodegenerative diseases. Interest in UA’s role in neurological disorders grew from its involvement in oxidative damage observed in Parkinson’s disease [104,105]. In HD, a secondary analysis of data from the CARE-HD clinical trial found that higher baseline UA levels were associated with slower progression of functional decline in HD, indicating that UA could be a valuable predictor of disease progression [106]. Moreover, Corey-Bloom et al. investigated UA levels in plasma and saliva from 38 HD patients, 31 preHD patients, and 38 normal controls, revealing significant gender differences. Male HD patients exhibited higher UA levels compared with females, with both plasma and salivary UA levels being significantly lower in female premanifest and manifest HD patients compared with controls. Salivary UA levels in male manifest HD patients were also notably lower than in controls. Gender-specific correlations were observed where UA levels in males negatively correlated with total functional capacity (TFC) and positively correlated with total motor score (TMS). In females, plasma UA levels positively correlated with TMS, and salivary UA levels correlated with disease burden. Additionally, decreased UA levels were found in postmortem prefrontal cortical samples from HD subjects [107]. These findings suggest that salivary UA could be a candidate non-invasive biomarker for HD, with variations in disease pathology being potentially influenced by sexual dimorphism.

24 (S) Hydroxycholesterol (24OHC)

24 (S) hydroxycholesterol (24OHC), a significant cholesterol metabolite in the brain, is notably reduced in the plasma of individuals with HD. This decrease correlates with a reduction in caudate volume [112], suggesting that 24OHC may serve as an indicator of progressive neuronal loss in HD [204]. Researchers have analyzed 24OHC levels in plasma from gene-expanded individuals and found notable differences across patient groups. Specifically, 24OHC concentrations decreased with advancing disease stages. The decrease in 24OHC was more substantial than changes in cognitive and motor function or neuroimaging alterations [109]. Gray et al. recently reported that lower plasma levels of 24OHC and altered 24OHC/25OHC ratios are associated with cognitive performance in early HD, suggesting that dysregulated cholesterol homeostasis may contribute to cognitive impairment in HD. While these findings highlight 24OHC as a potential biomarker, its utility may be limited by the specificity of associations to cognitive endpoints and the relatively small observed changes [205]. To validate this metabolite as a reliable biomarker, long-term monitoring of patients is suggested to track changes in metabolic markers throughout HD progression [204].

#### 3.2.3. Imaging Biomarkers

##### Magnetic Resonance Imaging (MRI)

Magnetic resonance imaging (MRI) offers a sophisticated approach for mapping brain atrophy, enabling precise visualization of structural changes that are pivotal for understanding neurodegenerative disorders. It is a non-invasive modality widely utilized to examine both structural and microstructural alterations in individuals with the Huntington’s disease (HD) gene, regardless of whether they are in the premanifest or manifest stages. Documented cases have shown that brain atrophy and structural changes occur before overt symptoms of HD become apparent [135,136]. MRI is used to reveal atrophy in the striatum and cortex of the brain correlated to HD progression. The increased size of the frontal horns of the lateral ventricles is another HD symptom that can be visualized by imaging. When MRI findings show strong correlations with clinical assessments, they could serve as more reliable indicators of disease progression, which is especially beneficial for large-scale, multicenter studies [137].

The most pronounced pathological changes in HD are observed in the striatum [138], with substantial evidence indicating that striatal atrophy can be detected by MRI up to 23 years before predicted motor symptoms appear [206]. Furthermore, striatal volume reductions are negatively correlated with both motor and cognitive functions, as well as with CAG repeat length [207]. Recently, reductions in cerebellar volume have been intricately linked to alterations in emotional functioning, symptom duration, and visuomotor performance. A cross-sectional study on a cohort of HD patients at different stages has revealed a decline in corticocerebellar functional connectivity in HD, with cerebellar atrophy correlating with gait disturbances, motor impairments, and deficits in emotion recognition and working memory [208,209,210]. However, to gain a more comprehensive understanding of the cerebellum’s role in HD, studies with larger sample sizes are imperative.

A study utilized 3T MRI to assess brain volumes, revealing significant reductions in whole-brain volume, as well as regional gray and white matter differences, in premanifest HD gene carriers with normal motor scores [178]. These structural changes precede clinical symptoms, highlighting the potential of neuroimaging as an early biomarker for HD. In the premanifest stage, MRI volumetry has emerged as a pivotal biomarker, facilitating early detection of structural changes even before the onset of clinical symptoms. This advancement has been instrumental in the development of the Huntington’s Disease Integrated Staging System (HD-ISS), which incorporates MRI volumetry to delineate disease progression from the earliest stages. HD-ISS introduces an early biomarker stage, underscoring the significance of MRI volumetry in monitoring individuals at risk for HD [10]. In summary, MRI volumetry serves as a critical tool in the early detection and staging of Huntington’s disease, offering valuable insights into the disease’s progression and aiding in the development of targeted therapeutic interventions.

##### Positron Emission Tomography (PET)

Positron emission tomography (PET) also offers a valuable insight into the disease progression [211]. Data from PET imaging indicates that changes in the brain of affected patients start years before the onset of the disease [36]. Glucose PET scans, utilizing techniques such as [18F] FDG PET and [11C] raclopride PET, are indispensable in the evaluation of HD. These imaging modalities quantify regional brain metabolism, yielding critical insights into the neurodegenerative processes underlying HD. A significant observation is that reduced metabolism in the caudate nucleus is correlated with bradykinesia and rigidity [139], diminished total functional capacity [212], and cognitive decline [213]. Moreover, decreased metabolic activity in the putamen is associated with impaired motor functions [139] and serves as a predictor of symptomatic conversion in individuals carrying the HD gene expansion [140]. Furthermore, PET imaging with the novel radioligand 11C-CHDI-180R has been validated for visualizing and quantifying mHTT aggregates, providing a promising tool for assessing mHTT load and evaluating the efficacy of therapeutic interventions in HD models [214]. Additionally, PET imaging using the radioligand [(18)F]CPFPX has revealed that cerebral adenosine receptor (A_1_AR) levels shift from supranormal levels in premanifest individuals to subnormal levels in manifest HD, correlating strongly with years to onset and highlighting its potential role in altered energy metabolism during conversion from the preHD to the manifest HD stage [215].

Monitoring putaminal metabolism could be crucial for early intervention strategies and for assessing the risk of symptom onset. Additionally, reduced metabolism in the striatum and cortex correlates with the duration and severity of chorea, the extent of overall disability [141], and the bicaudate ratio [142], an imaging marker indicative of caudate atrophy. These findings suggest that extensive metabolic decline in the striatum and cortex signifies advanced disease stages and greater levels of disability. Moreover, increased metabolism in the thalamus is linked to dystonia [139], while reduced metabolism in the thalamus is indicative of the overall disease [216].

Phosphodiesterase 10E (PDE10E), when assessed through PET tracers [18F] MNI-659 and [11C] IMA-107, provides critical insights into its role in HD. A cross-sectional study on a cohort of 11 HD and 9 healthy controls have shown that reduced PDE10E expression in the striatum and pallidum is associated with higher UHDRS motor scores, increased disease burden, and regional brain atrophy, indicating its contribution to motor dysfunction and structural changes observed in HD progression [217]. Furthermore, alterations in PDE10E levels, such as elevated levels in thalamic nuclei or diminished levels in the striato-pallidal projecting striatum, correlate with an increased likelihood of symptomatic conversion among HD gene carriers [218].

Studies have shown a significant decline in [11C] raclopride binding potential in early symptomatic individuals, but the reduction is not correlated with age of onset or disease duration, indicating a uniform process across HD stages [219]. Additionally, variability in imaging findings was noted by Antonini et al., where reductions in both [18F] FDG and [11C] raclopride uptake were observed in symptomatic patients, while asymptomatic gene carriers showed normal uptake with a gradual decline in [11C] raclopride binding over time [142]. Further studies, such as those by Van Oostrom et al. and Feigin et al., support these findings, showing normal initial uptake in preclinical carriers followed by gradual declines and highlighting the compensatory metabolic changes before symptom onset [220,221]. These results underscore the complexity of neuronal degeneration in HD, suggesting that functional imaging may be more effective when combined with other biomarkers for evaluating disease progression and preclinical changes.

##### Diffusion Tensor Imaging (DTI)

Diffusion tensor imaging (DTI) is the most commonly employed diffusion MRI method for evaluating neurodegenerative diseases [222]. It has been shown that in HD, DTI parameters are abnormal in the basal ganglia and corpus callosum. Specifically, fractional anisotropy (FA) is significantly increased in the caudate, putamen, and globus pallidus in both pre-symptomatic HD (pre-HD) and symptomatic HD (sym-HD) patients, while FA is significantly decreased in the corpus callosum compared with controls. Moreover, elevations in mean diffusivity (MD) were detected in the putamen and thalamus of both pre-symptomatic (pre-HD) and symptomatic Huntington’s disease (sym-HD) patients and in the caudate of symptomatic patients when compared with controls. Moreover, in symptomatic HD patients, radial diffusivity (RD) and axial diffusivity (AD) were markedly increased in the corpus callosum compared with controls [222,223].

Early studies, such as Weaver et al. [224], demonstrated significant longitudinal decreases in white matter FA and AD over a one-year period in a small cohort of HD subjects. However, other studies have shown discrepancies in these findings. For instance, Sritharan et al. [225] and Vandenberghe et al. [226] reported no significant longitudinal changes in mean diffusivity (MD) of the caudate, putamen, thalamus, and corpus callosum over similar or longer timeframes. A recent study by Odish et al. aligns with these later findings, showing significant cross-sectional differences but not longitudinal changes over a two-year period [227]. This discrepancy suggests that while early studies indicated measurable progression in diffusivity measures over short periods, more recent research with larger cohorts and longer durations may not detect these changes as reliably. Therefore, the sensitivity of DTI to capture small, progressive changes in HD over extended periods remains uncertain, and further research is needed to clarify these findings.

#### 3.2.4. Neuropsychological Tasks-Related HD Biomarkers

The PREDICT-HD study was a pivotal research effort aimed at understanding the early progression of HD before the onset of typical clinical symptoms. It involved mHTT gene-positive participants who did not yet meet the diagnostic criteria for HD. PREDICT-HD employed a broad range of assessments, including motor evaluations, psychiatric measures, and imaging techniques, to monitor early changes and predict disease onset and progression. The study found that changes across these markers could begin one to two decades before the predicted time of diagnosis, underscoring the value of early indicators for tracking disease progression. Notably, the PREDICT-HD study also investigated the CAG age product (CAP) score which, when integrated with other assessments, provided a robust framework for identifying individuals at risk of developing HD. In particular, the neuropsychological test battery used in the study demonstrated its value in capturing cognitive and sensory-perceptual changes, with composite measures offering greater sensitivity and predictive power than individual tests, highlighting the utility of this test battery in preventive HD trials and early interventions [228,229].

In clinical studies, tools like the HD-CAB (Huntington’s Disease Cognitive Assessment Battery), which was also developed for use in clinical trials, are especially relevant. HD-CAB incorporates cognitive tests, including those for verbal learning, emotion recognition, and motor planning, to assess cognitive decline across different stages of the disease. This composite measure, which has been shown to effectively differentiate between premanifest HD, early HD, and healthy controls, is an important advancement for monitoring cognitive dysfunction in HD [230]. Such neuropsychological measures are increasingly utilized alongside clinical scales like the UHDRS [231].

##### Motor Tapping

Motor abnormalities are a hallmark feature of HD. The manifestation, severity, and onset of these abnormalities are conventionally assessed using the UHDRS Total Motor Score (UHDRS-TMS). However, this scoring system is limited to HD gene carriers presenting clear motor symptoms [114,116]. Furthermore, UHDRS-TMS exhibits low sensitivity to subtle motor manifestations, leading to variability in individual assessments and challenges in the identification of disease onset in preHD individuals [114]. To address these limitations, the development of dedicated instruments for assessing the onset and severity of motor impairments in preHD individuals has been proposed [116]. In the premanifest stage, quantitative motor assessments, including voluntary neurophysiological motor tasks and oculomotor tasks, have identified impairments in HD gene carriers. However, after 24 months, despite significant declines in regional and overall brain volumes, only a few functional variables showed significant changes compared with controls. Notably, transcranial magnetic stimulation (TMS), emotion recognition, and speeded tapping were exceptions. This suggests that many neuropsychological biomarkers may not be sensitive enough in a longitudinal setting during the premanifest stage of HD [232]. The 2009 TRACK-HD study by Tabrizi et al. further explored the early biological and clinical manifestations of HD. This cross-sectional analysis of baseline data revealed significant reductions in brain volumes, including the striatum, and regional differences in gray and white matter in premanifest HD gene carriers, even with no motor symptoms. These findings support the potential of neuroimaging as an early biomarker for HD, showing that structural brain changes occur before clinical symptoms manifest, providing crucial insights into disease progression [233].

Quantitative tapping assessments have emerged as a sensitive biomarker for detecting early motor deficits and tracking disease progression in preHD patients [234]. A retrospective study evaluated the total number of taps performed by 237 HD individuals using a simple handheld tapping device to examine longitudinal disease progression [115]. The study demonstrated a linear decline in tapping performance, with a reduction of 5.1 taps per year and an additional decline of 5.6 taps associated with extra CAG repeats [5]. However, this study was conducted manually and included only individuals with manifest HD.

To enhance sensitivity, Antonaides et al. (2012) [235] conducted a pilot study employing simple tapping pads with touch sensors that functioned independently of applied force. This study recorded overall tapping rates and statistical characteristics of response intervals, identifying delayed tapping responses in manifest HD patients that correlated with traditional behavioral assessments. Nevertheless, the study was limited to individuals with manifest HD. A more comprehensive TRACK-HD study utilized force transducer-based tapping assessments across 120 preHD, 123 early symptomatic HD, and 123 control participants. This study revealed that the inter-onset interval (IOI) of tapping significantly differentiated preHD individuals from controls, with a maximum effect size of 1.03. Additionally, tapping frequency distinguished preHD from HD individuals, yielding an effect size of 0.71 [114]. The study further demonstrated a strong correlation between speeded tapping performance and brain volumetric changes, with preHD individuals exhibiting atrophy in the caudate and putamen and reduced cortical processing. These neurological changes impacted reflex execution and cognitive performance, as individuals with lower tapping rates showed declining results in neurophysiological assessments [236].

Additionally, digital motor biomarkers, such as remote digital monitoring platforms for assessing cognitive and motor symptoms in HD, offer continuous, real-time insights, further enhancing the sensitivity of motor impairment detection and disease progression tracking [237].

In conclusion, speeded tapping assessments represent a promising and reliable tool for early detection and monitoring of motor impairments in both premanifest and manifest Huntington’s disease. These assessments not only enhance sensitivity compared with traditional rating scales like the UHDRS-TMS but also provide a quantitative and non-invasive approach to evaluate disease onset, severity, and progression. By revealing subtle motor deficits and correlating with neurological changes, speeded tapping offers valuable insights into the underlying mechanisms of HD, supporting its potential for use in clinical trials and as a biomarker for early intervention.

##### Speech Biomarker

Dysarthric speech alterations represent one of the earliest clinical manifestations of HD, preceding severe motor, cognitive, and psychiatric symptoms [118,121]. Several cross-sectional studies have investigated speech changes using a range of acoustic measures [121]. Examples of these measures include diadochokinetic (DDK) rates for assessing speech articulation, maximum phonation time for respiratory capacity, hypernasality levels for resonance, and total speech time or silence ratio for evaluating prosody [121].

In one study, *HTT* gene-positive individuals showed slower speech rates, longer word production times, and increased silences between and within words. These speech changes significantly correlated with scores of disease burden, suggesting speech as an objective indicator of early HD onset and progression [238]. A cross-sectional study analyzing speech samples from 44 preHD individuals (29 presymptomatic and 15 prodromal), 25 manifest HD patients, and 25 matched controls identified significant alterations in speech. These changes included reduced articulation precision, impaired phonation, and disrupted speech timing in both prodromal and manifest HD individuals. Furthermore, the study established a correlation between the severity of speech impairments and slowed cognitive processing, motor deficits, and the CAG age product score, indicating that speech alterations are driven by underlying biological changes in the gene, even at the prodromal stage. Consequently, automated speech analysis has been proposed as a quantitative biomarker for the early detection of disease progression [120].

A multicenter prospective study further validated the utility of speech alterations as markers of disease progression. This study demonstrated that changes in rhythmic and articulatory speech features are indicative of progressive disease status, motor and cognitive decline, and reduced striatal volume in HD patients. Using a predictive machine learning model, the study achieved error rates of 12% to 20% in forecasting functional, motor, and cognitive decline in HD individuals [239]. Additionally, a cross-sectional study involving 28 preHD individuals and 28 matched healthy controls identified compromised steadiness and regularity in syllable repetition, beginning at the preHD stage [123]. Interestingly, preHD individuals exhibited an increased speech pace compared with controls, interpreted as dysfunctional hyper-compensation linked to increased gray matter volume and reduced basal ganglia integrity [121,123]. Therefore, speech alterations are among the earliest detectable biomarkers of HD progression. These findings underscore the potential of speech-based metrics, combined with advanced computational models, to provide sensitive and non-invasive tools for tracking disease onset and progression.

##### Digital Biomarker

Digital biomarkers are emerging as powerful non-invasive tools in HD, offering objective and continuous data to monitor disease progression. These biomarkers leverage advanced technologies, including speech analysis, wearable sensors, and imaging modalities, to capture subtle changes in motor, cognitive, and behavioral domains, often before clinical symptoms manifest [240]. For instance, the Roche HD digital monitoring platform exemplifies the application of digital biomarkers by integrating smartphone- and smartwatch-based tools for active and passive symptom monitoring. This system demonstrated strong correlations between digital measures (e.g., speech and motor performance) and established clinical scales like the UHDRS. These findings highlight the potential of digital biomarkers for remote tracking of disease progression and early interventions [237].

Speech-based digital biomarkers have shown significant promise in detecting early HD-related changes. Advanced tools like BioDigit Speech software have been utilized to analyze speech patterns, focusing on features such as pausing, intelligibility, and timing. Studies have demonstrated that speech features differ between HD patients, prodromal individuals, and healthy controls, correlating with clinical scores and disease burden. Machine learning models trained on speech data have achieved impressive accuracy in predicting disease severity, highlighting the potential of speech analysis as an early, quantitative marker of HD progression [117,118,241].

Wearable and portable sensors enhance the ability to track HD progression remarkably. Devices equipped with accelerometers and gyroscopes provide detailed data on movement patterns, sleep disturbances, and circadian rhythms. These sensors allow for real-time, continuous monitoring of motor symptoms such as bradykinesia and chorea, which are critical for understanding disease dynamics. In addition, the portability of these devices facilitates remote patient monitoring, expanding access to care and enabling earlier interventions. However, the lack of standardized methodologies remains a challenge, necessitating efforts to harmonize protocols for broader applicability [118].

Digital biomarkers provide sensitive and scalable solutions for tracking disease onset, progression, and treatment response. Their integration into clinical workflows has the potential to revolutionize personalized care for HD patients, but further research is needed to address existing limitations, such as standardization and validation across diverse populations [133].

##### EEG and fMRI

Electroencephalography (EEG) is a non-invasive method that measures brain oscillatory activity changes, reflecting synaptic dysfunction and progressive neurodegeneration in Huntington’s disease (HD). There have documented changes in EEG power among patients with manifest HD, notably an increase in delta power and a decrease in alpha power [132,242,243]. In individuals with premanifest HD (preHD) and early manifest HD (EMHD), there has been a reported reduction in power within the low alpha band and at the theta–alpha border [242,244,245]. Moreover, research has shown that the number of CAG repeats in the HTT gene correlates with EEG changes and cognitive decline in both preclinical and early manifest HD patients [244,246,247]. A recent cross-sectional observational study aimed to identify neurophysiological alterations in preclinical and early manifest HD by analyzing EEG and fMRI resting-state functional connectivity (rsFC) and examining their interrelationships. This study demonstrated that EEG and fMRI can effectively reveal neurophysiological changes associated with HD. Specifically, it found decreased power in certain EEG frequency ranges in both preclinical (preHD) and early manifest (EMHD) stages compared with healthy controls. These EEG changes were linked with disrupted functional connectivity in fMRI analyses, particularly within frontal, putamen-cortical, and cortico-cerebellar networks. The findings suggest that integrating EEG and fMRI provides valuable insights into HD, with decreased alpha and theta–alpha power correlating with increased connectivity in large-scale brain networks, which may contribute to cognitive decline [131]. While these findings are promising, the utility of EEG as a reliable biomarker for HD remains under investigation and requires critical evaluation. For instance, EEG was used as a biomarker in the first antisense oligonucleotide study targeting m*HTT* expression, but the results were not considered significant for further discussion [248]. Furthermore, more specific EEG tasks, such as integrating event-related potentials (ERPs) with structural MRI data and source localization using sLORETA, have been proposed. These multi-methodological approaches, particularly those focusing on fronto-striatal networks, may offer greater sensitivity for monitoring striatal pathology. Studies like those by Beste et al. [130,249] underscore the potential of these methods, emphasizing their relevance in examining fronto-striatal synchronization processes for response inhibition and action monitoring. Further research is warranted to establish EEG’s clinical utility and its applicability in HD.

##### Event Related Potentials (ERPs)

Event-related potentials (ERPs) are time-locked electrophysiological responses to specific sensory, cognitive, or motor events, measured via electroencephalography (EEG). They provide a non-invasive window into the temporal dynamics of neural processing, capturing the brain’s immediate reactions to stimuli. ERPs are characterized by distinct components, such as the N100 and P300, each associated with different stages of information processing [125]. The N100 component, for instance, is linked to early sensory processing, while the P300 component is associated with attention and stimulus evaluation processes. The amplitude and latency of these components offer insights into cognitive functions, including attention, memory, and decision-making. For example, variations in the P300 component have been utilized to study cognitive decline in neurodegenerative diseases as well as to assess cognitive load in human–computer interaction contexts [126].

Event-related potentials (ERPs) have emerged as valuable biomarkers in Huntington’s disease (HD), offering insights into the cognitive and neural alterations associated with the condition. Studies have demonstrated that HD patients exhibit significant changes in ERP components, such as prolonged latencies and reduced amplitudes, indicating deficits in cognitive processing and attentional mechanisms. These ERP abnormalities correlate with the severity of motor and cognitive symptoms, suggesting their potential utility in monitoring disease progression [127]. Moreover, ERPs have been employed to assess the efficacy of therapeutic interventions in HD, providing objective measures of treatment-related changes in neural function. The non-invasive nature and high temporal resolution of ERPs make them promising tools for the early detection and ongoing assessment of HD, complementing other neuroimaging and clinical evaluations [250].

**Figure 7:**
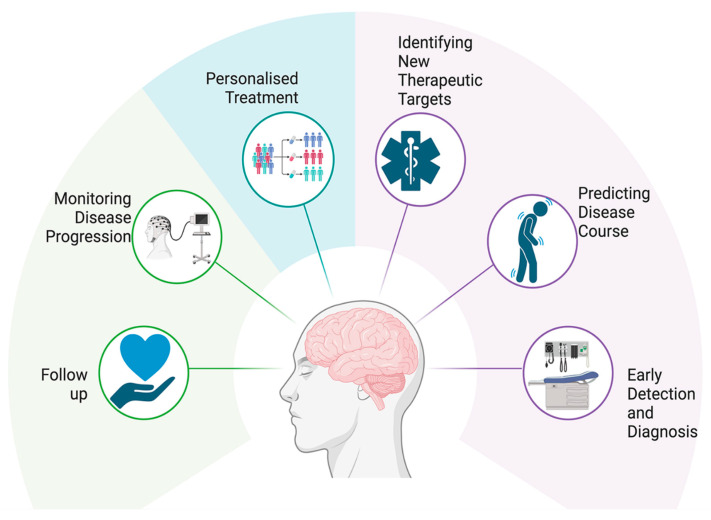
The role of biomarkers in Huntington’s disease.

Recent studies have further explored the role of ERPs in HD. For example, research has indicated that HD patients may exhibit increased cognitive functioning in certain tasks, as revealed by behavioral and ERP indices of auditory sensory memory and attention [128]. Additionally, combined studies using ERPs and voxel-based morphometry have provided insights into error processing levels in HD, highlighting the complex neural alterations associated with the disease [129]. Furthermore, a study examining EEG functional connectivity in premanifest and manifest HD patients found increased phase synchronization across multiple frequency bands, correlating with cognitive decline. This suggests that EEG connectivity measures, including those derived from ERP components, could serve as potential biomarkers for early phenotypical expression and disease progression in HD [243]. Expanding on this, Beste et al. (2013) developed a novel cognitive-neurophysiological biomarker by integrating ERP components with behavioral measures in premanifest HD individuals. This biomarker demonstrated sensitivity to disease progression over six months, surpassing the predictive value of traditional parameters. The study highlighted the potential of combining ERP data with behavioral assessments to track early neural and cognitive changes in HD. Such integrative approaches underscore the utility of ERPs not only in detecting neurophysiological alterations but also in providing comprehensive tools for monitoring disease progression and evaluating therapeutic intervention [251]. Figure 7 highlights how these biomarkers function across various stages of HD, emphasizing their significance in understanding and managing the disease.

## 4. Discussion

Huntington’s disease poses a serious threat to individuals globally, imposing significant economic and social burdens on nations due to its high management costs and complex symptoms. This highlights the critical need for research into HD to improve understanding and treatment of the disease. The development of fast and reliable methods for diagnosis, progression monitoring, and therapeutic success evaluation of neurodegenerative disorders is a key research domain. Recent genetic studies suggest that Huntington’s disease may be more prevalent than previously estimated, with underdiagnosis and incomplete penetrance contributing to discrepancies in reported prevalence. Findings highlight a higher frequency of disease alleles in the population, underscoring the need for improved diagnostic accuracy and broader awareness across diverse populations [252]. Biomarkers serving as indicators of disease encompass genetic markers, neuroimaging markers, and biofluid markers, such as proteins, immunological markers, and microRNAs found predominantly in blood and cerebrospinal fluid (CSF) [70]. Early detection of NDs in general and of HD in particular is crucial for the evaluation of targeted medications that may delay the disease progression [100]. A diagnosis of HD in patients with a family history is usually straightforward; however, around 8% of the patients do not have an affected family member, which requires quick and reliable diagnostic tools [6]. Biomarkers extend beyond diagnostic purposes: they are also designed for prognostic, predictive, and staging applications. Throughout the disease’s progression, biomarkers can detect its presence, assess severity, monitor its advancement, and evaluate treatment responses [100].

While CSF has traditionally been considered the most reliable biofluid for biomarker collection, the difficulty in its collection methods has led researchers to develop blood-based biomarkers, which offer a faster and easier evaluation process. Although blood-based biomarkers have shown promise, further research is needed to refine their accuracy and understand the factors affecting their levels before they can be routinely used in clinical laboratories [70]. This study represents the first bibliometric analysis linking biomarkers with Huntington’s disease research, offering a comprehensive overview of research trends over the past decade.

The analysis identified 730 relevant articles published between 2014 and 2024, a substantial number that reflects significant research activity in this area. The substantial number of publications highlights a robust and ongoing commitment to advancing biomarker research, which is vital for improving the diagnosis and management of Huntington’s disease. Early detection of neurodegenerative diseases like Huntington’s disease is essential for evaluating therapies that might slow disease progression, with biomarkers playing key roles in diagnosis, prognosis, staging, and monitoring treatment responses [100]. A keyword analysis in this study revealed that terms associated with other neurodegenerative diseases, such as Parkinson’s disease (PD), Alzheimer’s disease (AD), and amyotrophic lateral sclerosis (ALS), were less frequently used in the context of HD research. This is somewhat surprising, given that HD shares several pathological and clinical features with these disorders [253]. The relatively lower emphasis on these related conditions suggests an area of potential growth in HD research. Exploring the relationships between HD and other neurodegenerative diseases could yield important insights, particularly in identifying common biomarkers or therapeutic targets that span multiple disorders.

The average of seven co-authors per article highlights the collaborative nature of research in this field. Such collaboration is essential as it brings together diverse perspectives and expertise, which are crucial for addressing the complex challenges associated with biomarker discovery and validation. The increase in publication numbers, particularly the surge observed in recent years, further highlights the growing recognition of the importance of biomarkers in HD research [36]. This trend suggests that the scientific community is increasingly focused on developing biomarkers that can lead to more targeted therapies and better patient outcomes.

The analysis also reveals a significant gap in international collaboration, particularly with countries in Africa. The lack of research could be partly attributed to insufficient medical evaluation such as molecular testing and limited access to comprehensive knowledge on variable HD symptoms. These factors limit the physician’s ability to provide and record an appropriate diagnosis. Furthermore, lack of adequate medical assistance such as supportive care and specialized nursing facilities often leads to HD patients succumbing to the disease at early stages. These challenges could contribute to a lower HD prevalence in the African subcontinent when compared with the European counterpart [41]. The lack of research contributions from this region is concerning as it limits the global understanding of HD. While it is possible that the lower prevalence of HD in Africa contributes to this disparity [3], it indicates the need for more inclusive research efforts that involve diverse populations. Greater collaboration with researchers from underrepresented regions could provide valuable insights and enhance the generalizability of biomarker findings.

The neurofilament light chain (NfL) stands out as a crucial biomarker, and it is extensively validated for its ability to reflect disease progression and neurodegeneration through quantifiable measures in cerebrospinal fluid (CSF) and plasma [75,76,79]. The utility of mutant huntingtin (mHTT) protein levels, particularly in CSF, also remains significant for distinguishing between different stages of HD [69,70,179]. Emerging biomarkers show considerable promise in enriching our understanding and diagnosis of HD: these include microRNAs (miRNAs), tau protein, and 24(S)-hydroxycholesterol (24OHC), each contributing unique insights into HD’s pathophysiology [93,98,205]. Additionally, motor tapping and speech analysis have gained traction as functional biomarkers that can effectively monitor motor and cognitive declines in HD patients. These functional biomarkers provide direct, measurable insights into patient capabilities and disease impact over time. Coupled with advances in neuroimaging techniques such as MRI and PET, which offer structural and metabolic perspectives well before symptom onset [135,211,219], the integration of these diverse biomarkers within a multi-biomarker strategy enhances the overall diagnostic accuracy and monitoring of HD, addressing its complex symptomatology encompassing motor, cognitive, and psychiatric dimensions. A multi-biomarker strategy could significantly enhance the accuracy of HD diagnosis and monitoring, addressing the diverse motor, cognitive, and psychiatric symptoms that characterize the disease. By combining genetic, neuroimaging, and biofluid biomarkers, this approach could provide a more comprehensive view of disease progression, improving diagnostic sensitivity and offering personalized treatment options. Integrating these biomarkers into clinical practice could enable more effective disease management by identifying individuals at risk, monitoring disease progression, and evaluating therapeutic responses [240]. Future directions in HD biomarker research involve several key areas. First, conducting longitudinal studies is crucial to tracking disease progression and treatment outcomes over extended periods, ensuring the reliability and clinical relevance of identified biomarkers [44]. Second, extensive research efforts should be focused on exploring emerging biomarkers aimed at deepening our understanding of disease mechanisms and enhancing diagnostic accuracy. Third, encouraging collaboration among researchers and clinicians globally can accelerate biomarker discovery and validation processes [240]. By standardizing protocols and ensuring reproducibility across diverse study populations and settings, these initiatives aim to advance personalized approaches to effectively managing Huntington’s disease. By addressing these areas, the scientific community can work toward a more inclusive and comprehensive understanding of HD, ultimately leading to better diagnostic tools, treatment options, and patient outcomes worldwide.

## 5. Study Limitations

This bibliometric analysis has some limitations that should be acknowledged. A primary limitation is the restriction to articles sourced solely from the PubMed database, which may narrow the scope of the findings. Expanding the search to include other databases, such as Scopus or Web of Science, could provide a more comprehensive and nuanced view of the research landscape. Another limitation is the exclusion of non-English publications, which may have led to the omission of valuable insights from studies conducted in other languages and cultural contexts. Additionally, the reliance on bibliometric data does not account for the quality or impact of the publications analyzed, which could influence the interpretation of the findings. Lastly, while the analysis provides a quantitative overview, it does not delve deeply into the specific methodologies or findings of individual studies, which may limit its applicability in guiding specific research directions.

The field of HD biomarker research is advancing at a remarkable pace, making it challenging to comprehensively capture all recently discovered biomarkers. While this study provides an updated review of the literature on HD biomarkers, it acknowledges this limitation. This highlights the critical need for continuous updates in this field to ensure that newly discovered biomarkers are accurately documented and effectively integrated into the existing body of knowledge.

## 6. Conclusions

In conclusion, this study offers a comprehensive review and bibliometric analysis of HD biomarker research, highlighting the substantial number of publications and the evident collaboration among researchers in this field. However, the lack of representation from certain geographical regions and the limited exploration of connections with other neurodegenerative diseases reveal important gaps that warrant future attention. Addressing these disparities can enhance the global understanding of HD and improve outcomes for HD patients.

Despite advancements, identifying reliable biomarkers for HD remains a significant challenge due to HD heterogeneous presentation, encompassing diverse and variable motor, cognitive, and psychiatric symptoms. Longitudinal observational studies have provided valuable insights into disease progression through imaging techniques and clinical tests, identifying key neurological, behavioral, motor, and cognitive alterations [229,254,255,256]. However, these efforts have yet to yield consistent and dependable HD biomarkers.

Huntington protein involvement in multiple biological pathways—including protein aggregation, neuroinflammation, and mitochondrial dysfunction—complicates biomarker identification. Moreover, factors beyond the primary HTT gene mutations, such as genetic modifiers and environmental influences, add more variability to disease onset, progression, and presentation, further complicating the identification of reliable biomarkers. Therefore, HD presents a multifaceted pathology, underscoring the challenge of finding a single biomarker that accurately reflects the disease process and necessitating the use of a panel of HD biomarkers [44].

These complexities are exacerbated by difficulties in recruiting and retaining patients for long-term studies. Such studies are critical for validating potential biomarkers and ensuring their clinical relevance. Moving forward, focused efforts on addressing these challenges through collaborative research, innovative methodologies, and inclusive study designs will be essential for advancing HD biomarker discovery and improving clinical outcomes.

## Figures and Tables

**Table 1:** Search analysis summary.

Description	Results
Timespan	2013:2024
Sources (Journals, Books, etc.)	319
Documents	730
Annual Growth Rate %	9.13%

## Data Availability

Data are contained within the article.
